# Delineation of the role of chromatin assembly and the Rtt101^Mms1^ E3 ubiquitin ligase in DNA damage checkpoint recovery in budding yeast

**DOI:** 10.1371/journal.pone.0180556

**Published:** 2017-07-27

**Authors:** Li-Ting Diao, Chin-Chuan Chen, Briana Dennehey, Sangita Pal, Pingping Wang, Zie-Jie Shen, Angela Deem, Jessica K. Tyler

**Affiliations:** 1 Department of Pathology and Laboratory Medicine, Weill Cornell Medicine, New York, New York, United States of America; 2 Department of Epigenetics and Molecular Carcinogenesis, University of Texas, MD Anderson Cancer Center, Houston, Texas, United States of America; Dana Farber Cancer Institute, UNITED STATES

## Abstract

The DNA damage checkpoint is activated in response to DNA double-strand breaks (DSBs). We had previously shown that chromatin assembly mediated by the histone chaperone Asf1 triggers inactivation of the DNA damage checkpoint in yeast after DSB repair, also called checkpoint recovery. Here we show that chromatin assembly factor 1 (CAF-1) also contributes to chromatin reassembly after DSB repair, explaining its role in checkpoint recovery. Towards understanding how chromatin assembly promotes checkpoint recovery, we find persistent presence of the damage sensors Ddc1 and Ddc2 after DSB repair in *asf1* mutants. The genes encoding the E3 ubiquitin ligase complex Rtt101^Mms1^ are epistatic to *ASF1* for survival following induction of a DSB, and Rtt101^Mms1^ are required for checkpoint recovery after DSB repair but not for chromatin assembly. By contrast, the Mms22 substrate adaptor that is degraded by Rtt101^Mms1^ is required for DSB repair *per se*. Deletion of *MMS22* blocks loading of Rad51 at the DSB, while deletion of *ASF1* or *RTT101* leads to persistent Rad51 loading. We propose that checkpoint recovery is promoted by Rtt101^Mms1^-mediated ubiquitylation of Mms22 in order to halt Mms22-dependent loading of Rad51 onto double-stranded DNA after DSB repair, in concert with the chromatin assembly-mediated displacement of Rad51 and checkpoint sensors from the site of repair.

## Introduction

DNA double-strand breaks (DSBs) occur often, arising on average ten times per cell per day [1]. While DSBs are common, they are the most deleterious of genotoxic lesions, as they can result in translocations if misrepaired and loss of chromosomal segments if unrepaired. Accordingly, the cell has developed multiple pathways to try to ensure the accurate repair of DSBs and maintain genomic integrity. DSB repair pathways fall into two main classes. One class is non-homologous end joining (NHEJ) which requires no sequence homology and is potentially mutagenic [2]. The other class is homologous recombination (HR), which precisely repairs DSBs via the use of an undamaged homologous sequence elsewhere in the genome [3].

Much of our knowledge of eukaryotic DSB repair comes from studies in budding yeast, where an inducible HO endonuclease is placed under the control of a galactose-inducible promoter, in order to efficiently create a single DSB at a defined genomic location [4]. From this system, we know that an early event during all DSB repair pathways is the 5’ to 3’ resection of the DNA ends to yield 3’ single-stranded DNA (ssDNA) [3]. SsDNA is bound by the ssDNA binding protein RPA [5]. During HR, RPA is later removed and replaced with Rad51, and together with Rad52, they promote the strand invasion step that is required to complete the homology search [6, 7]. Single strand annealing (SSA) is a variant of HR that is used to repair a DSB that is flanked by two identical sequences and uses a subset of the HR machinery. SSA does not require strand invasion but instead the DNA resection reveals the identical ssDNA sequences, which anneal together and any remaining non-complementary ssDNA tails are clipped off by Rad1 [8].

In parallel to the repair of the DNA molecule, an intricate signaling cascade is activated called the DNA damage checkpoint that promotes DNA repair and cell cycle arrest. In budding yeast, the DNA damage cell cycle checkpoint is under the control of the kinase Mec1, the ATR homolog [9]. The ssDNA coated by RPA is independently recognized by the Mec1-Ddc2 and Rad17-Mec3-Ddc1 checkpoint sensor complexes [10–12], where Rad17-Mec3-Ddc1 activates Mec1 in response to DSBs [13, 14]. Activated Mec1 leads to the phosphorylation of Rad53, a homolog of human CHK1, which is the central checkpoint effector kinase in cell cycle arrest in yeast [15, 16]. The cell cycle remains arrested until after the DNA lesion is repaired. Exactly how the cell senses that DNA repair is complete is not clear, but once this happens, it results in inactivation of the cell cycle checkpoint, also called checkpoint recovery [17].

In yeast, checkpoint recovery is studied using SSA repair assay systems where the regions of homology are on the same chromosome but 5-30kb apart, because the resection over this long distance ensures activation of the DNA damage checkpoint [18]. Checkpoint recovery is accompanied by the disappearance of phosphorylated Rad53 from the cell, which is achieved in part by the redundant action of the phosphatases Pph3, Ptc2, and Ptc3. However, the Rad53 phosphatases only partially contribute to full checkpoint recovery [19]. Presumably, checkpoint recovery must also involve disengagement of the checkpoint sensor complexes Mec1-Ddc2 and Rad17-Mec3-Ddc1 from the site of DNA repair to prevent further phosphorylation of Rad53, but how this occurs is unclear. Another protein that is required for checkpoint recovery is the helicase Srs2 [18]. Biochemically, Srs2 removes Rad51 from ssDNA [20, 21]. Mechanistically, Srs2 is required to displace Rad51 from the ssDNA tails that exist after DNA annealing during SSA [22]. The Rad51 that persists at the site of DSB repair upon deletion of *SRS2* is accompanied by the persistent presence of Ddc2, and presumably also the Mec1 kinase, at the site of DNA repair [22], explaining why the checkpoint fails to recover in the *srs2* mutant.

Within the cell, DNA repair has to occur in the context of chromatin. The basic repeating unit of chromatin, termed the nucleosome, is made up of two molecules each of histone H2A, H2B, H3, and H4 with approximately 147 base pairs of DNA wrapped around this histone octamer [23]. By its very nature, chromatin provides a formidable obstacle to the repair machinery gaining access to the DNA lesion. We showed previously that chromatin is disassembled around a DSB during DNA repair and that chromatin is reassembled after DSB repair in yeast [24]. Unexpectedly, we found that assembly of chromatin over the site of DSB repair was required for checkpoint recovery [24]. Although Asf1 is a histone chaperone for H3/H4 [25], Asf1 does not deposit the histones onto the repaired DNA *per se*, but instead promotes acetylation of histone H3 lysine 56 (H3 K56Ac) [26], which drives chromatin assembly after repair [24]. The likely mechanism for how H3 K56Ac drives chromatin assembly after repair probably depends on the higher affinity of H3 K56Ac for the replication-dependent histone chaperone chromatin assembly factor 1 (CAF-1) [27] that is physically tethered to sites of DNA synthesis through its interaction with PCNA [28]. However, it has not yet been shown that CAF-1 mediates chromatin assembly after DSB repair in yeast. Given that chromatin assembly would not occur until after DSB repair is complete, conceptually, the assembly of chromatin provides an elegant mechanism to inform the cell that DNA repair is complete. But how chromatin assembly signals to the checkpoint machinery to trigger checkpoint recovery is unclear.

In this work, we further investigate how chromatin is assembled after DSB repair and how chromatin assembly signals to the checkpoint machinery to turn off the DNA damage checkpoint after DSB repair. We show that CAF-1 contributes to the assembly of chromatin after DSB repair. Blocking chromatin assembly after DSB repair led to the persistent presence of the checkpoint sensors Ddc1 and Ddc2 at the site of repair. We also find that the E3 ubiquitin ligase Rtt101^Mms1^ is epistatic to Asf1, being required for checkpoint recovery but not for chromatin assembly after DSB repair. Meanwhile, the Mms22 adaptor protein, which is also a polyubiquitylation substrate of the Rtt101^Mms1^ complex [29] is required for DSB repair *per se*, via its requirement for loading of Rad51, reminiscent of the scenario in human cells [30, 31]. Mutants that fail to assemble chromatin after DSB repair or that fail to degrade Mms22 led to the persistent presence of Rad51 at the repaired DNA. We propose that the concerted action of chromatin assembly and inactivation of Mms22 after DNA repair promote the displacement of Rad51, Ddc1 and Ddc2 to enable checkpoint recovery.

## Materials and methods

### DNA repair analyses

**Fig 1:**
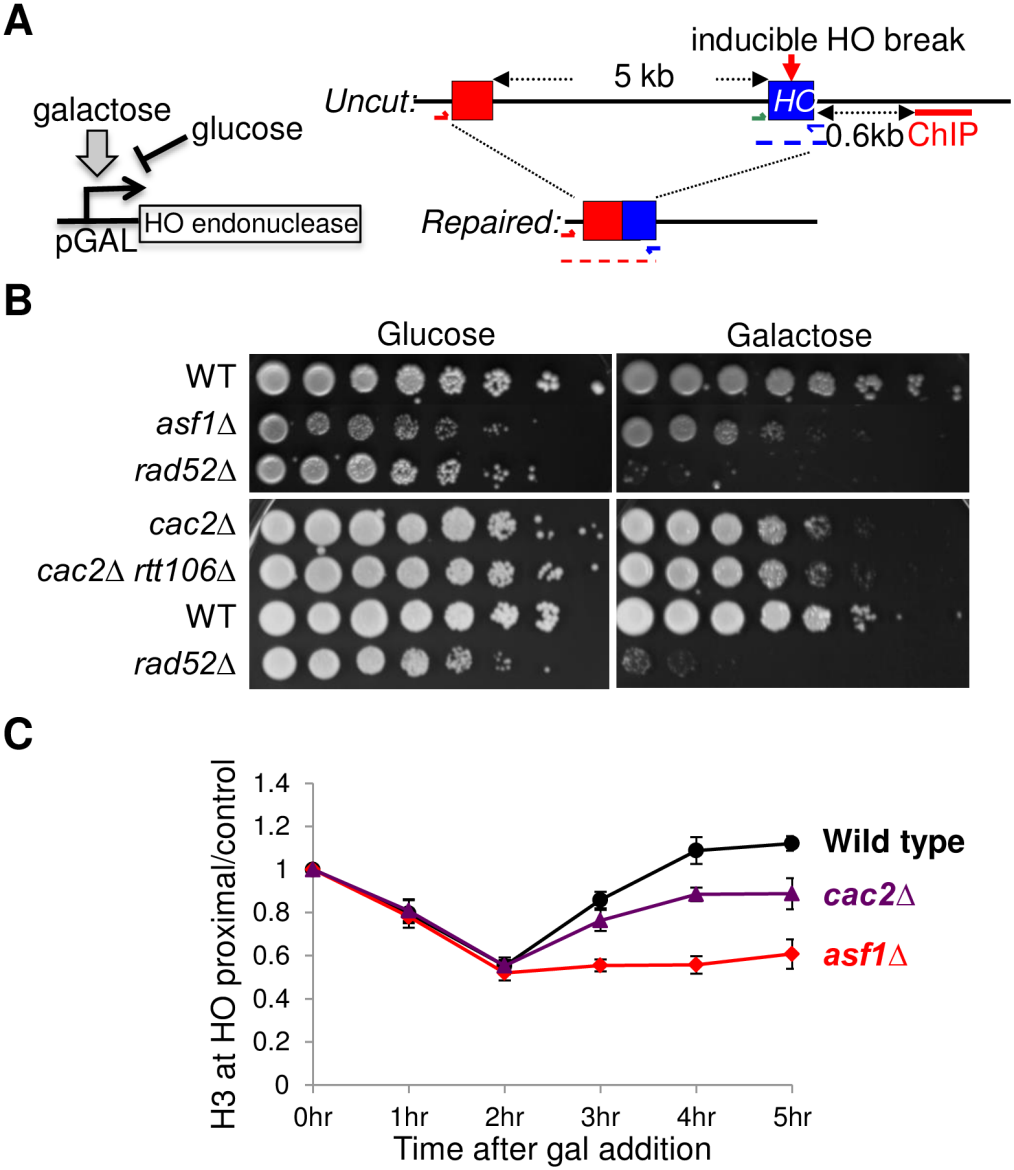
CAF-1 but not Rtt106 is involved in chromatin assembly after DSB repair. **A**. Experimental system for measuring SSA. The galactose inducible HO endonuclease system induces one specific DSB in a defined location (red arrow) in order to analyze the DSB repair *in vivo* by PCR analysis with the indicated primer pairs. Repair of the HO lesion at the HO cleavage site (blue box) requires 5kb of resection back to the uncleavable HO cleavage site (red box). **B**. 10-fold serial dilution analysis of the indicated isogenic yeast strains WT (YMV045), *asf1*Δ (JKT200), *rad52*Δ (YMV046), *cac2*Δ (JLY078), and *cac2*Δ *rtt106*Δ (CCY020) to show the sensitivity to a single HO lesion. The WT data in the top panel are from the same petri dish as the other strains, but sections of the photograph were rearranged to obtain an optimal order of the strains for the figure. **C**. ChIP analysis of H3 levels flanking the DSB site (0.6kb) in WT (YMV045), *asf1*Δ (JKT200) and *cac2*Δ (JLY078) strains. The HO lesion was induced by adding galactose at 0hr. The H3 ChIP data were normalized to a control region on another chromosome (*SMC2*). All data are the average and standard deviation of three independent experiments.

Cultures were diluted down to OD_600_ of ~0.3 in YEPR and allowed to grow back for at least 4hrs until the cells reached an optical density of approximately OD_600_ 0.5. Cells were plated in 10-fold serial dilutions onto YPD or rich media + 2% raffinose + 2% galactose (YEPG). After 2–3 days of growth, the cells were photographed to record colony formation. Alternatively 2% galactose was added to log phase cultures growing in YEPR and samples were taken for cutting and repair, western, or ChIP analyses at the time points indicated in the Figures; the 0hr samples were taken prior to addition of galactose. Cutting and repair of the HO site in the single strand annealing strains was performed using the three primers indicated in Fig 1A, that we described previously [24]. PCR analysis prior to repair yields a 1.7kb PCR product, during DNA damage yields no PCR product, and following repair by SSA yields a 3.0kb product. Primers to the *RAD3* gene were included in the multiplex PCR as an internal control. The number of PCR cycles to produce amplification in the linear range was determined empirically. Primer pairs for analysis of SSA cutting and repair: Control primer pair: RAD3A:GATAAGATTGCGACAAAAGAGGATA; RAD3D:GTGGGACGAGACGTTTAGATAGTAA. HO flanking primer set: SSA1:CCGCTGAACATACCACGTTG; SSA2:CACTTCCAGATGAGGCGCTG; SSA3:TGAACTCTGGTGTCTTTTAG.

### Chromatin immunoprecipitation analyses

Cells were crosslinked with formaldehyde, followed by shearing of the chromatin, immunoprecipitation and quantitation of the DNA sequences in the immunoprecipitates via real time PCR analysis, as previously described [24]. Chromatin assembly and disassembly were measured via ChIP analyses using an antibody to the C-terminus of H3 (Abcam #1791), H3 K56Ac (Upstate #07–677) or Rad51 antisera (Abcam #63798). Plotted are the average and standard deviation of the mean of three independent experiments. Primer pairs / probe sequences for ChIP analysis of HO site at MAT:

SMC2 primer pair: F-GGTCCGGTAAGTCGAACATTTT; R-CTCGCACAGTGCTCATTGATG. 0.6kb HO primer pair: F-TTGGATCTTAACAAACCGTAAAGGT; R-GGTAACTAGCAAACAAAGGAAAGTCA Primer pairs for ChIP analysis of HO site during SSA: For the HO Cut Site: 5': CCAAATCTGATGGAAGAATGGG; 3': CCGCTGAACATACCACGTTG. For the control on telomere VI Right Arm: 5': GGATTTTACCAACGACTTCGTCTCA; 3': CGCTATTCCAGAAAGTAGTCCAGC

### Western blot analysis

Protein from mid-log phase culture was isolated by TCA (trichloroacetic acid) precipitation and resolved by electrophoresis on a SDS-polyacrylamide gel. The following primary antibodies were used for Immunoblotting: anti-HA (16B12; Covance), anti-Rad53 (EL7; gift from Pellicioli lab / Abcam, #104232), anti-Myc (Santa Cruz Biotechnology, sc-789), anti-GAPDH (Sigma, A-9521), anti tubulin (AbD serotec, MCA78G), anti-phosphorylated Rad53 (F9; gift from Pellicioli lab), anti-H3 (Abcam #1791), and anti-Ptc2 (Rabbit serum, gift from Dr. Marie-Claude Marsolier-Kergoat).

### Microscopic recovery analyses

Individual unbudded G1 cells were generated by starvation and sonication as described by [24]. These cells were then spread onto plates containing YEPR + 2% galactose to induce the HO lesion, and the number of cells/buds of the same region was assayed using a dissection microscope at the times indicated in the figures.

### Flow cytometry

Cells from each time points were fixed in ethanol and treated with RNase A for 1 hour at 37°C, followed by treatment with proteinase K for 1 hour at 50°C, and next stained with propidium iodide as described previously [32]. Cells were briefly sonicated immediately before being scanned with a Beckman Coulter XL-MCL machine. DNA content reflects an average of at least 10,000 cells.

### Yeast strains used are listed in Table 1

**Table 1:** Yeast strains.

Name	Genotype
JKT200	MAT α ho hml::ADE1 mata::hisG hmr::ADE1 leu2(Asp-718-SalI)-URA3-pBR322-HOcs ade3::GAL::HO ade1 lys5 ura3-52 asf1::kan [24]
YMV045	MATα ho hml::ADE1 mata::hisG hmr::ADE1 leu2(Asp-718-SalI)-URA3-pBR322-HOcs ade3::GAL::HO ade1 lys5 ura3-52 [18]
YMV046	MATα ho hml::ADE1 mata::hisG hmr::ADE1 leu2(Asp-718-SalI)-URA3-pBR322-HOcs ade3::GAL::HO ade1 lys5 ura3-52 rad52::HPH [18]
CCY018	MATα ho hml::ADE1 mata::hisG hmr::ADE1 leu2(Asp-718-SalI)-URA3-pBR322-HOcs ade3::GAL::HO ade1 lys5 ura3-52 mms1::KAN This study
CCY019	MATα ho hml::ADE1 mata::hisG hmr::ADE1 leu2(Asp-718-SalI)-URA3-pBR322-HOcs ade3::GAL::HO ade1 lys5 ura3-52 rtt101::KAN This study
CCY020	MATα ho hml::ADE1 mata::hisG hmr::ADE1 leu2(Asp-718-SalI)-URA3-pBR322-HOcs ade3::GAL::HO ade1 lys5 ura3-52 rtt106::KAN cac2::Hygro This study
CCY022	MATα ho hml::ADE1 mata::hisG hmr::ADE1 leu2(Asp-718-SalI)-URA3-pBR322-HOcs ade3::GAL::HO ade1 lys5 ura3-52 rtt107::KAN This study
CCY024	MATα ho hml::ADE1 mata::hisG hmr::ADE1 leu2(Asp-718-SalI)-URA3-pBR322-HOcs ade3::GAL::HO ade1 lys5 ura3-52 mms22::KAN This study
CCY026	MATα ho hml::ADE1 mata::hisG hmr::ADE1 leu2(Asp-718-SalI)-URA3-pBR322-HOcs ade3::GAL::HO ade1 lys5 ura3-52 asf1::KAN rtt101::HIS This study
CCY027	MATα ho hml::ADE1 mata::hisG hmr::ADE1 leu2(Asp-718-SalI)-URA3-pBR322-HOcs ade3::GAL::HO ade1 lys5 ura3-52 asf1::KAN mms1::HIS This study
JLY078	MATα ho hml::ADE1 mata::hisG hmr::ADE1 leu2(Asp-718-SalI)-URA3-pBR322-HOcs ade3::GAL::HO ade1 lys5 ura3-52 cac2::Hygro This study
LWY003	MATa ade2-1 can1-100 his3-11 leu2-3, 112 trp1-1 ura3-1 GAL pGAL-HO::ADE3 asf1::HYGRO RTT101-13Myc::TRP1 This study
LWY012	MATa ade2-1 can1-100 his3-11 leu2-3, 112 trp1-1 ura3-1 GAL pGAL-HO::ADE3 RTT101-13Myc::TRP1 This study
LWY016	MATa his3Δ1 leu2Δ0 met15Δ0 ura3Δ0 his5+::pGAL::3xHA-MMS22 This study
LWY023	MATα ho hml::ADE1 mata::hisG hmr::ADE1 leu2(Asp-718-SalI)-URA3-pBR322-HOcs ade3::GAL::HO ade1 lys5 ura3-52 MMS22-3xHA::TRP1 This study
LWY033	MATa his3Δ1 leu2Δ0 met15Δ0 ura3Δ0 his5+::pGAL::3xHA-MMS22 rad52::KANMX6 This study
LWY061	ho hmlΔ::ADE1 mataΔ::hisG hmrΔ::ade1 leu2::leu2(Asp-718-SalI)-URA3-pBR322-HOcs ade3::GAL::HO ade1 lys5 ura3-52 trp1 RAD1::KANMX6
CFY046	MATa his3Δ1 leu2Δ0 met15Δ0 ura3Δ0 his5+::pGAL::3xHA-MMS22 rtt101Δ::URA3
CFY039	MATa his3Δ1 leu2Δ0 met15Δ0 ura3Δ0 his5+::pGAL::3xHA-MMS22 asf1Δ::URA3
ptc2Δ	MATa his3Δ1 leu2Δ0 met15Δ0 ura3Δ0 ptc2Δ::KANMX

## Results

### CAF-1 contributes to the assembly of chromatin after DSB repair

We had previously shown that yeast lacking the H3-H4 histone chaperone CAF-1 are sensitive to DNA double-strand damaging agents even though they have no defect in repair of DSBs *per se* [33]. Given that CAF-1 assembles chromatin after nucleotide excision repair [34], we considered it a good candidate to test for a role in chromatin assembly after DSB repair and checkpoint recovery. Yeast mutants with defects in checkpoint recovery show sensitivity to growth on galactose-containing media, which induces an HO lesion that is repaired by SSA [18] (Fig 1A). Using the SSA system, a *cac2* mutant, where *CAC2* encodes a subunit of CAF-1, showed sensitivity to induction of the HO endonuclease, albeit less so than an *asf1* mutant, suggesting that CAF-1 plays a role in survival after DSB repair (Fig 1B). Rtt106 is a histone chaperone that contributes to chromatin assembly after DNA replication in a redundant manner with CAF-1 [27]. If this were also the case following DSB repair, the *rtt106 cac2* double mutant would be more sensitive than a *cac2* mutant to the single HO break. However, the *rtt106 cac2* double mutant was equally sensitive as the *cac2* mutant (Fig 1B), indicating that Rtt106 is unlikely to be mediating chromatin assembly and checkpoint recovery after DSB repair. To determine whether CAF-1 does indeed contribute to chromatin assembly following DSB repair, we analyzed the histone H3 levels adjacent to the HO lesion by chromatin immunoprecipitation (ChIP) analysis. As we have shown previously, the kinetics of chromatin assembly followed the kinetics of DSB repair in wild type yeast, while chromatin disassembly failed to occur in the *asf1* mutant despite efficient repair [24] (Fig 1B and S1 Fig). Similarly, the *cac2* mutant was competent at DSB repair, yet had a defect in chromatin reassembly after DSB repair (Fig 1C and S1 Fig). This is consistent with the weak DNA damage sensitivity seen with the *cac2* mutant, as compared to the *asf1* mutant (Fig 1B). In agreement, efficient DSB repair of the HO lesion at the *MAT* locus in yeast lacking Asf1 or CAF-1 was also observed previously [24] [33] [35]. As such, we conclude that CAF-1 contributes to chromatin assembly after DSB repair, although presumably another H3-H4 histone chaperone may also receive histones from Asf1 to mediate the remainder of the chromatin reassembly after DSB repair. The role of CAF-1 in chromatin assembly after DSB repair likely explains the contribution that CAF-1 makes to checkpoint recovery after DSB repair [35].

### A transient patch of H3 K56Ac exists at the site of repair

**Fig 2:**
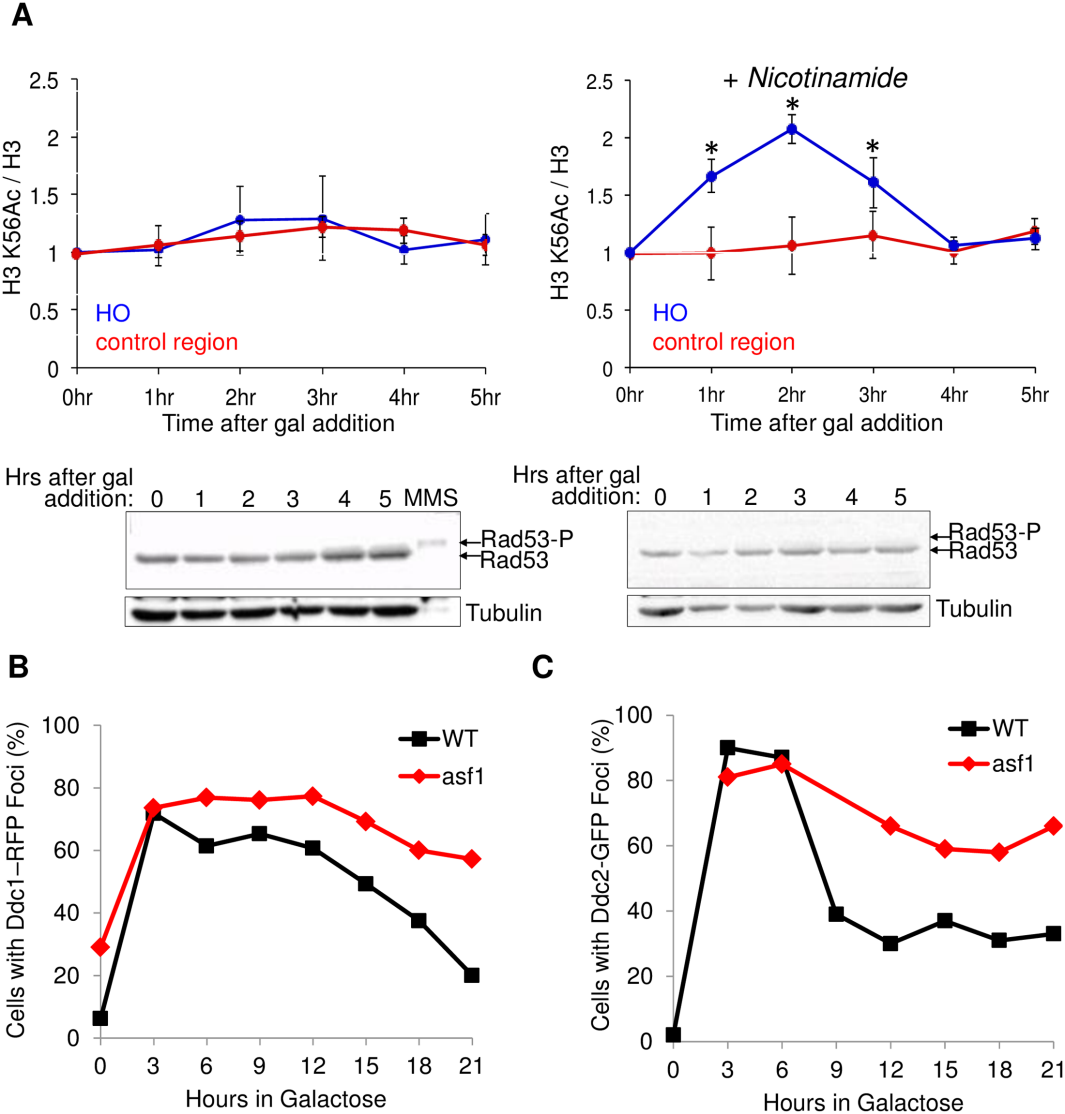
Blocking chromatin assembly leads to persistent checkpoint sensor presence at the site of repair. **A**. The top panels show the histone H3 K56Ac levels assessed by ChIP analysis adjacent to the DSB site (0.6Kb) and at a control region *SMC2* in a wild type strain (YMV045). The H3 K56Ac ChIP data were normalized to H3 levels and to the input. All data are the average and standard deviation of three independent experiments. Asterisk (*) indicates significant changes compared to time 0 (p<0.05), as determined by the Student’s t-test. The lower panels show immunoblotting for the checkpoint kinase Rad53, where activation of the checkpoint leads to phosphorylation of Rad53 (Rad53-P) apparent as a slower migrating species. Tubulin serves as a loading control. Right panels (+Nicotinamide) are as in left panels, but in the presence of 25 mM nicotinamide. **B**. Analysis of proportion of cells with Ddc1-RFP in repair foci or **C**. Ddc2-GFP in repair foci at the indicated times following addition of galactose. Over 100 cells were examined at each time point. The data shown are from one experiment and are representative of the typical results.

Asf1 promotes chromatin assembly after DSB repair via its role in acetylating H3 K56Ac, as evidenced by the ability of an H3 K56Q mutant to bypass the requirement for Asf1 in chromatin assembly after DSB repair [24]. This result implied that histones carrying the H3 K56Ac mark would be assembled at the site of DSB repair. To test whether this was the case, we asked whether there was local enrichment of H3 K56Ac at the site of DSB repair by ChIP analysis. Even though this region of the genome is already transcribed and presumably has H3 K56Ac before DNA damage induction, we found that H3 K56Ac levels increased upon induction of the HO endonuclease at the site of DSB repair, but not at a control region, but only when we included the class III histone deacetylase inhibitor nicotinamide (Fig 2A). This result demonstrates that there is a local patch of H3 K56Ac at the site of DSB repair, but that it is very rapidly deacetylated. Even with the presence of nicotinamide, the H3 K56Ac in the region of the DSB disappeared with increasing time after DNA repair, consistent with the dynamic histone exchange that occurs throughout the yeast genome that others and we have reported previously [36, 37].

There are several possible models to explain why Asf1, and the H3 K56Ac that it mediates, is required for checkpoint recovery: 1. Chromatin assembly *per se* could trigger checkpoint recovery, for example by displacing a repair and / or checkpoint protein from the site of DSB repair. 2. The transient patch of H3 K56Ac at the site of DSB repair could trigger checkpoint recovery by recruiting a H3 K56Ac binding protein that is involved in checkpoint recovery. 3. The deacetylation of H3 K56Ac at the site of chromatin assembly could lead to the dissociation of an H3 K56Ac binding protein from the site of DSB repair, in order to trigger checkpoint recovery. Model 1 and 2 are not experimentally separable, given that H3 K56Ac is required for chromatin assembly after DSB repair [24]. To address model 3, we asked whether prolonging the existence of the patch of H3 K56Ac at the site of DSB repair, by addition of nicotinamide, leads to persistent checkpoint activation. In the presence of nicotinamide we saw no detectable increase in phosphorylation or induction of the Rad53 checkpoint kinase, which is diagnostic for checkpoint activation (Fig 2A). As such, it would appear that H3 K56Ac deacetylation is not required for checkpoint recovery *per se*.

The Rad53 phosphatases Ptc2 and Pph3 are known to contribute to checkpoint recovery [38, 39]. One potential explanation for the contribution of Asf1 and Rtt101/Mms1 to checkpoint recovery could be via indirectly regulating the levels of these phosphatases. As such, we asked if the levels of the Rad53 phosphatases were reduced in yeast lacking Asf1 or Rtt101, and found that was not the case (S2 Fig and data not shown).

### Retention of Ddc1 and Ddc2 at the site of repair occurs when chromatin assembly is blocked

Our previous molecular evidence for persistent activation of the DNA damage checkpoint after DSB repair when we disrupted chromatin assembly was in the form of prolonged existence of phosphorylated Rad53 and cell cycle arrest [24]. To determine if loss of phosphorylated Rad53 was the only defective step in checkpoint recovery or whether the recovery of additional upstream events in the DNA damage pathway was also disrupted, we examined recruitment of the checkpoint sensors Ddc1 and Ddc2 to the DSB. When we examined recruitment of Ddc1 to the HO site undergoing SSA repair via foci analysis, we consistently observed in multiple independent experiments persistent retention of Ddc1 in the *asf1* mutant compared to wild type (Fig 2B). We also observed a similar retention of Ddc2 in repair foci after repair of the HO lesion by SSA in the *asf1* mutant (Fig 2C). The kinetics of appearance and disappearance of Ddc1 and Ddc2 at repair foci is similar to what has been seen previously using this same SSA repair system in the wild type yeast [22]. The Ddc1 and Ddc2 persisted at the site of repair in the *asf1* mutant up to 14 hours after DNA repair, given that repair is only delayed by approximately one hour in the *asf1* mutant, most likely due to delayed expression of the HO endonuclease (see later and [24]). These results indicate that a proportion of the checkpoint sensor proteins remain at the site of repair after repair is complete, when we block chromatin reassembly.

### The E3 ubiquitin ligase Rtt101^Mms1^ is required for checkpoint recovery after induction of a single DSB

**Fig 3:**
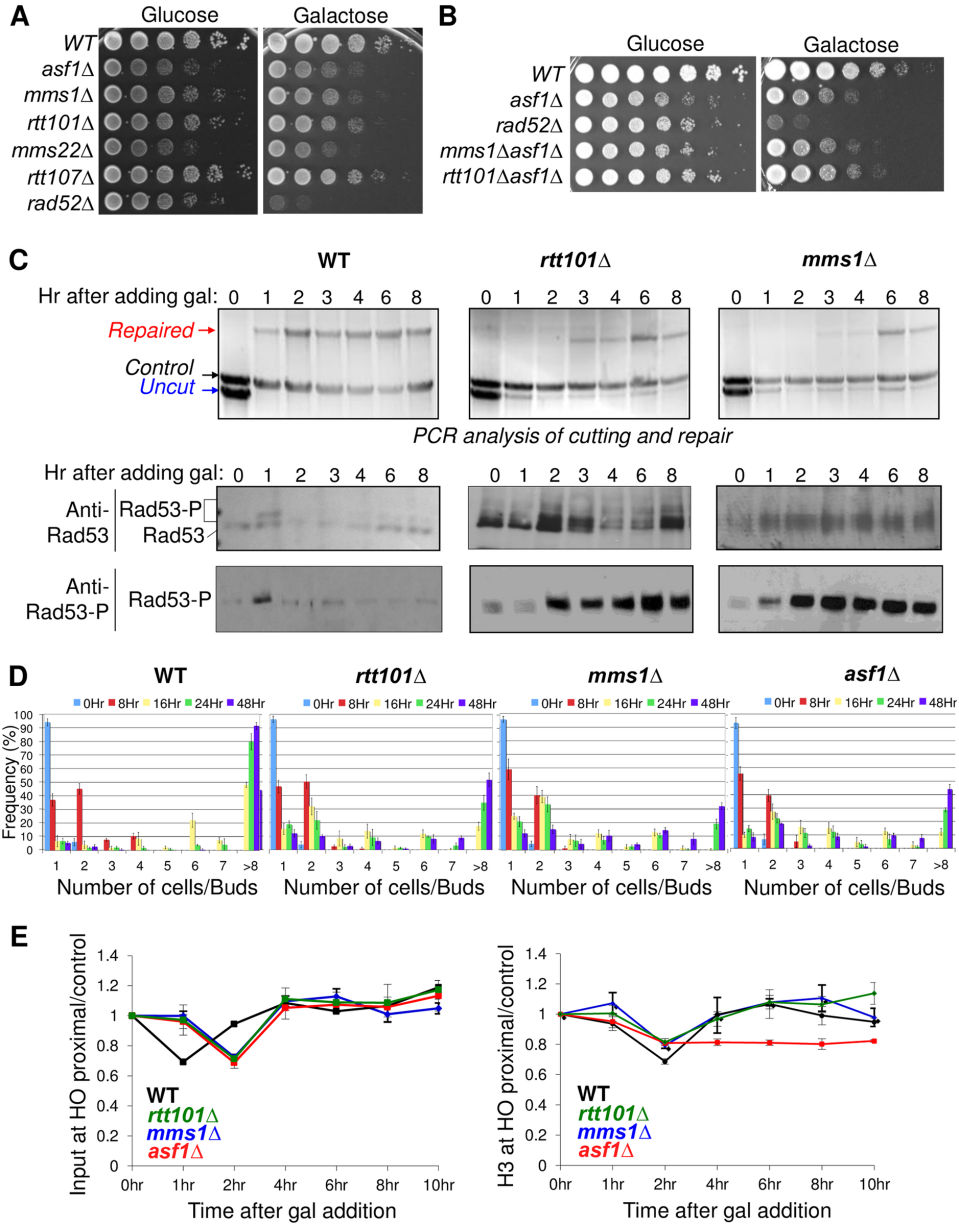
Rtt101^Mms1^ ubiquitin ligase functions in the same pathway as Asf1 in response to a DSB. **A and B**. 10-fold serial dilution analysis of WT (YMV045), *asf1*Δ (JKT200), *rad52*Δ (YMV046), *rtt101*Δ (CCY019), *mms1*Δ (CCY018), *mms22*Δ (CCY024), *rtt107*Δ (CCY022), *asf1*Δ *rtt101*Δ (CCY026), and *asf1*Δ *mms1*Δ (CCY027) strains containing the SSA HO repair system was performed as described in Fig 1B. **C**. The HO endonuclease was induced by addition of galactose at 0hr in the WT (YMV045), *rtt101*Δ (CCY019), and *mms1*Δ (CCY018) strains. The top panels show an analysis of the cutting and repair. The lower panels show immunoblotting for the checkpoint kinase Rad53 from the same time course as the repair analysis, using a pan Rad53 antisera. The panels at the bottom show immunoblotting for phosphorylated Rad53 only. **D**. Calculation of colony formation from single unbudded cells following the indicated length of times of growth on galactose-containing plates in WT (YMV045), *rtt101*Δ (CCY019), *mms1*Δ (CCY018) and *asf1*Δ (JKT200) strains. Error bars represent standard deviation calculated from three independent experiments. **E**. Analysis of DNA levels (input) and H3 levels flanking the HO lesion using the identical strains shown in **D**. The left panel shows the input, and the right panel shows the ChIP analysis of H3. Both input and ChIP data were normalized as in Fig 1C. All data are the average and standard deviation of three independent experiments.

Epistasis miniarray profile (MAP) analysis had previously indicated that Asf1 and the H3 K56Ac histone acetyl transferase Rtt109 were genetically upstream of the Rtt101^Mms1^ Mms22 E3 ubiquitin ligase complex for growth [40]. Therefore, we asked whether Rtt101^Mms1^ Mms22 were also functioning in the same pathway as Asf1 for checkpoint recovery. We found that *rtt101* and *mms1* mutants were sensitive to induction of the HO lesion that is repaired by SSA, while the *mms22* mutant was very sensitive (Fig 3A). Furthermore, the *mms1asf1* and *rtt101asf1* double mutants were as sensitive as the single *asf1* mutant, indicating that they function in the same pathway for survival after DSB repair (Fig 3B). Rtt101^Mms1^ Mms22 have been implicated previously in replication restart along with Rtt107 [41]. However, we find that *rtt107* mutants are not sensitive to the induction of the HO lesion that is repaired by SSA (Fig 3A) suggesting that Rtt107 is not involved in checkpoint recovery after DSB repair. Next, we analyzed whether Rtt101^Mms1^ were required for SSA repair of the DNA lesion. Using the PCR assay of cutting and repair [24], we found that the *rtt101* and *mms1* mutants were capable of DSB repair (Fig 3C). The repair in these mutants was delayed compared to the wild type, but the induction of the break was also delayed compared to wild type. Regardless, the extent of DNA repair was similar to wild type at the six-hour time point in the WT, *rtt101* and *mms1* mutants (Fig 3C). By contrast, activation of the DNA damage checkpoint, as determined by phosphorylation of Rad53, was seen only in the 1 hour time point in the wild type strain, yet persisted through to the last time point (8 hours) in the *rtt101* and *mms1* mutants (Fig 3C). These results indicate that Rtt101^Mms1^ contribute to checkpoint recovery after induction of a single DSB.

To determine unequivocally whether Rtt101^Mms1^ is required for checkpoint recovery, we examined the ability of cells to resume cell division after DNA damage. To do this, we measured the effect of inducing the HO lesion on the ability of single cells to divide over time, as previously described [18] (Fig 3D). As a control we included the *asf1* mutant that has a known role in checkpoint recovery [24]. Following induction of the DSB, 90% of the wild type cells divided twice by 48 hours, while around 30–50% of the *asf1*, *rtt101*, or *mms1* mutant had divided twice by 48 hours. Importantly, this result was not due to the*se* mutants having a growth defect on galactose plates, because growth of the mutants resembled wild type on galactose plates in strains where the HO site cannot be cleaved (data not shown). Taken together, we conclude that Rtt101 and Mms1 are involved in checkpoint recovery.

Next we asked whether Rtt101^Mms1^ is required for chromatin assembly after DSB repair, because chromatin assembly is known to be required for checkpoint recovery [24]. By histone H3 ChIP analysis, chromatin reassembly was equivalent in the wild type, *rtt101* and *mms1* mutants adjacent to the HO break that was repaired by SSA (Fig 3E). As a control, we included the *asf1* mutant, which does no chromatin assembly after DSB repair (Fig 3E). Efficient DNA repair in the absence of Rtt101^Mms1^ or Asf1 was also apparent in the ChIP input samples (Fig 3E), showing again that while HO cutting is delayed in the *mms1* and *rtt101* mutants, DNA repair occurs as effectively as wild type, albeit with a similar delay to the delay in DNA cutting. Because Rtt101^Mms1^ is not required for chromatin assembly, we propose that Rtt101^Mms1^ contributes to checkpoint recovery at a step downstream of chromatin assembly after DSB repair.

### Overexpression of MMS22 is not sufficient to cause persistent activation of the cell cycle checkpoint after DNA damage

**Fig 4:**
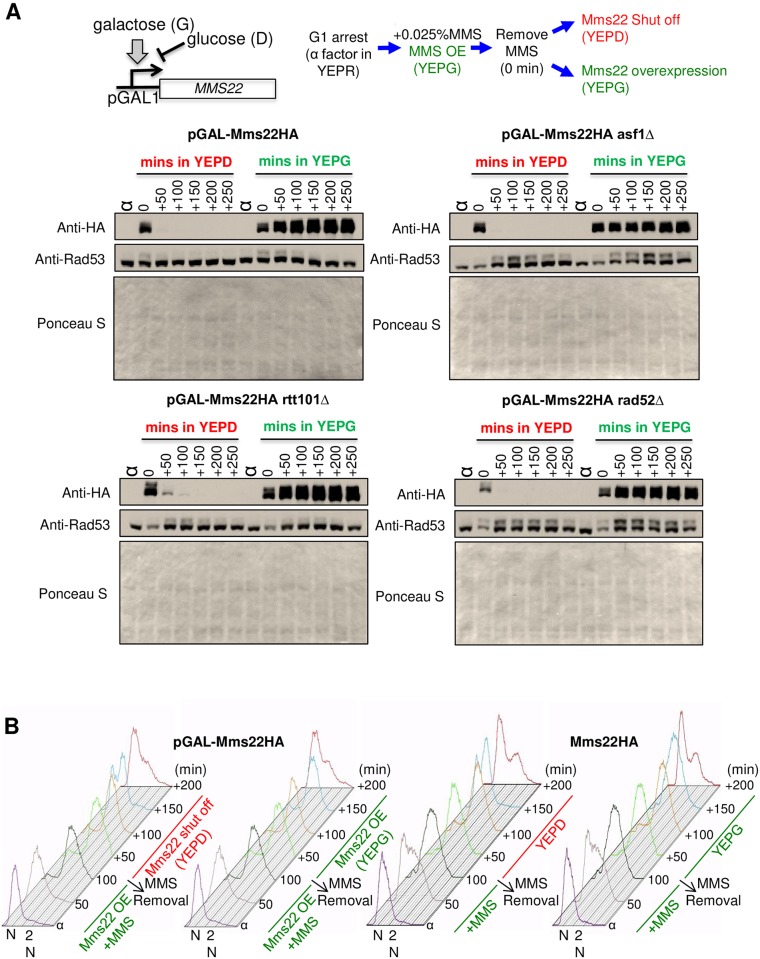
Transcriptional repression of Mms22 is insufficient to bypass the role of Asf1 or Rtt101 in checkpoint recovery. **A**. Experimental flowcharts. Yeast strains containing pGAL1-Mms22HA were arrest at G_1_ phase with alpha factor and released into YPEG (overexpressed Mms22HA) plus 0.025% MMS for 100 minutes. MMS was washed out and cells were allowed to recover in either YEPG (overexpressed Mms22HA) or YEPD (shut off Mms22HA) medium. Below is shown the levels of Mms22HA and Rad53 as measured by western blotting. The ponceau S is of the same membrane used for the westerns to show equal total protein loading, where the top half was used for probing for HA and the bottom half for probing for Rad53. The strains used were: GALMms22HA (LWY016), *rad52*Δ (LWY033), *asf1*Δ (CFY039) and *rtt101*Δ (CFY046). **B**. Analysis of cell cycle distribution following the same schematic shown in A, but measuring cell cycle phase by flow cytometry. Strains used were pGAL1-Mms22HA (LWY016), and Mms22HA (LWY023).

An earlier study had concluded that Rtt101^Mms1^-mediated degradation of its substrate adaptor Mms22 enhanced checkpoint recovery following exposure to the DNA methylating agent MMS [29]. Specifically, using Mms22 under the control of the galactose inducible *GAL1* promoter (pGAL1), they showed that transcriptional repression (followed by degradation) of *MMS22* after DNA repair, could overcome the DNA damage checkpoint arrest, while overexpression of Mms22 led to persistent DNA damage checkpoint arrest upon treatment with MMS [29]. Therefore, we took the same approach to determine whether transcriptional repression of pGAL1Mms22 could bypass the requirement for Rtt101 or Asf1 in checkpoint recovery after DNA damage. By contrast to expectation from the earlier study, we found that checkpoint arrest, as indicated by phosphorylation of Rad53, persisted upon Mms22 removal from the cell, in both the *rtt101* and *asf1* mutants (Fig 4A). Furthermore, we observed that persistent expression of Mms22, while it delayed checkpoint recovery in the wild type, this delay in recovery was only by approximately an hour (Fig 4A). We also wondered whether the previously reported delay of checkpoint recovery upon overexpression of Mms22 [29] was an indirect consequence of culturing the cells in galactose to induce Mms22, given that cells grow slower in galactose. Accordingly, we found that cells expressing pGAL-Mms22HA or Mms22HA under the endogenous promoter, showed the identical degree of cell cycle delay after DNA repair when grown in galactose, irrespective of whether the Mms22 had been removed from the cell or not (Fig 4B). We interpret these experiments to indicate that the use of chimeric promoters mediating overexpression and repression of Mms22 is unlikely to be a good mimic of localized Mms22 stabilization or degradation specifically at the site of repair in the cell.

### Mms22 promotes loading of Rad51 at the site of DSB repair

**Fig 5:**
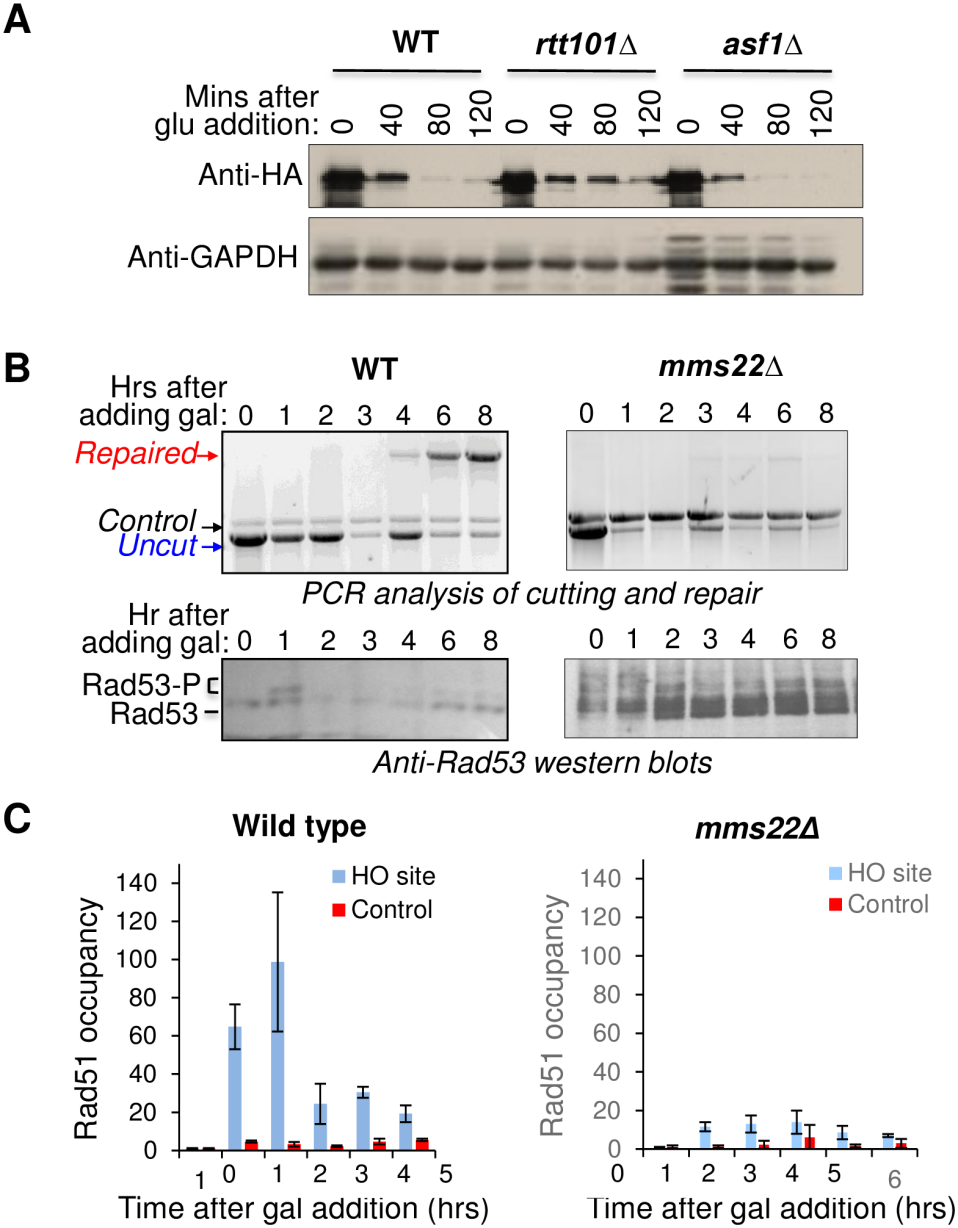
Mms22 promotes DSB repair via loading of Rad51. **A**. Analysis of stability of Mms22-HA at the indicated times after shutting off transcription of pGAL1-Mms22HA by addition of glucose to cultures previously grown in YEPG. Strains used are wild type pGAL1-Mms22HA (LWY016), *asf1*Δ (CFY039) and *rtt101*Δ (CFY046). **B**. The HO endonuclease was induced by addition of galactose at 0hr in the WT (YMV045) and *mms22*Δ (CCY024) strains. The top panels show an analysis of the cutting and repair. The lower panels show immunoblotting for the checkpoint kinase Rad53 from the same time course as the repair analysis. **C**. ChIP analysis of Rad51 levels flanking the DSB site and a TELVIR control region in WT (YMV045) and *mms22*Δ (CCY024) strain. Data are normalized to the input. Data are the average and standard deviation of three independent experiments.

As another approach to examine the relationship amongst the Rtt101^Mms1^ Mms22 complex and Asf1, we asked whether Asf1 is involved in its recruitment to DNA or displacement of the complex from DNA. When we examined relative levels of Rtt101 on chromatin versus off chromatin following induction of global DNA damage with the radiomimetic zeocin, we did not see any detectable difference in Rtt101 levels in the presence or absence of Asf1 (S3 Fig). These data indicate that Asf1 is not involved in recruitment of Rtt101^Mms1^ Mms22 to DSBs in yeast. We then asked whether Asf1 is required for the ubiquitylation and subsequent degradation of Mms22 by Rtt101^Mms1^. We found that the half-life of a portion of Mms22 was significantly increased in the absence or Rtt101, but not in the absence of Asf1 (Fig 5A). As such, Asf1 is not required either for recruitment of Rtt101^Mms1^ to sites of DSB repair or for degradation of Mms22.

Next, we examined whether Mms22, like Rtt101^Mms1^, was required for checkpoint recovery after DSB repair. However, we found that the situation with the *mms22* mutant was quite different from Rtt101^Mms1^. The *mms22* mutant had a clear defect in DSB repair (Fig 5B). The *mms22* mutant also demonstrated persistent Rad53 phosphorylation (Fig 5B) and checkpoint arrest after DSB induction (S4 Fig), likely due to the failure to repair the DNA damage (Fig 5B).

We wanted to better understand how the persistent presence of Mms22, presumably at the site of DSB repair, in the *rtt101* mutant would be deleterious to checkpoint recovery. In mammalian cells, knockdown of Mms22 leads to the persistent presence of RPA in the vicinity of DSBs [30, 31], leading to the proposal that Mms22 may mediate the unloading of RPA from ssDNA or loading of Rad51 onto ssDNA. Therefore, we asked if there was also a defect in Rad51 loading in the absence of Mms22 in yeast. By ChIP analysis, we found that Rad51 is recruited to the site of DNA repair in wild type yeast, peaking 2 hours after induction of the HO endonuclease (Fig 5C). By contrast, deletion of *MMS22* blocked recruitment of Rad51 to the vicinity of the HO lesion (Fig 5C). As such, Mms22 is required, directly or indirectly, to promote Rad51 loading at the site of DSB repair in yeast.

### Persistent presence of Mms22 or inhibition of chromatin assembly leads to persistent Rad51 bound over the site of DSB repair

**Fig 6:**
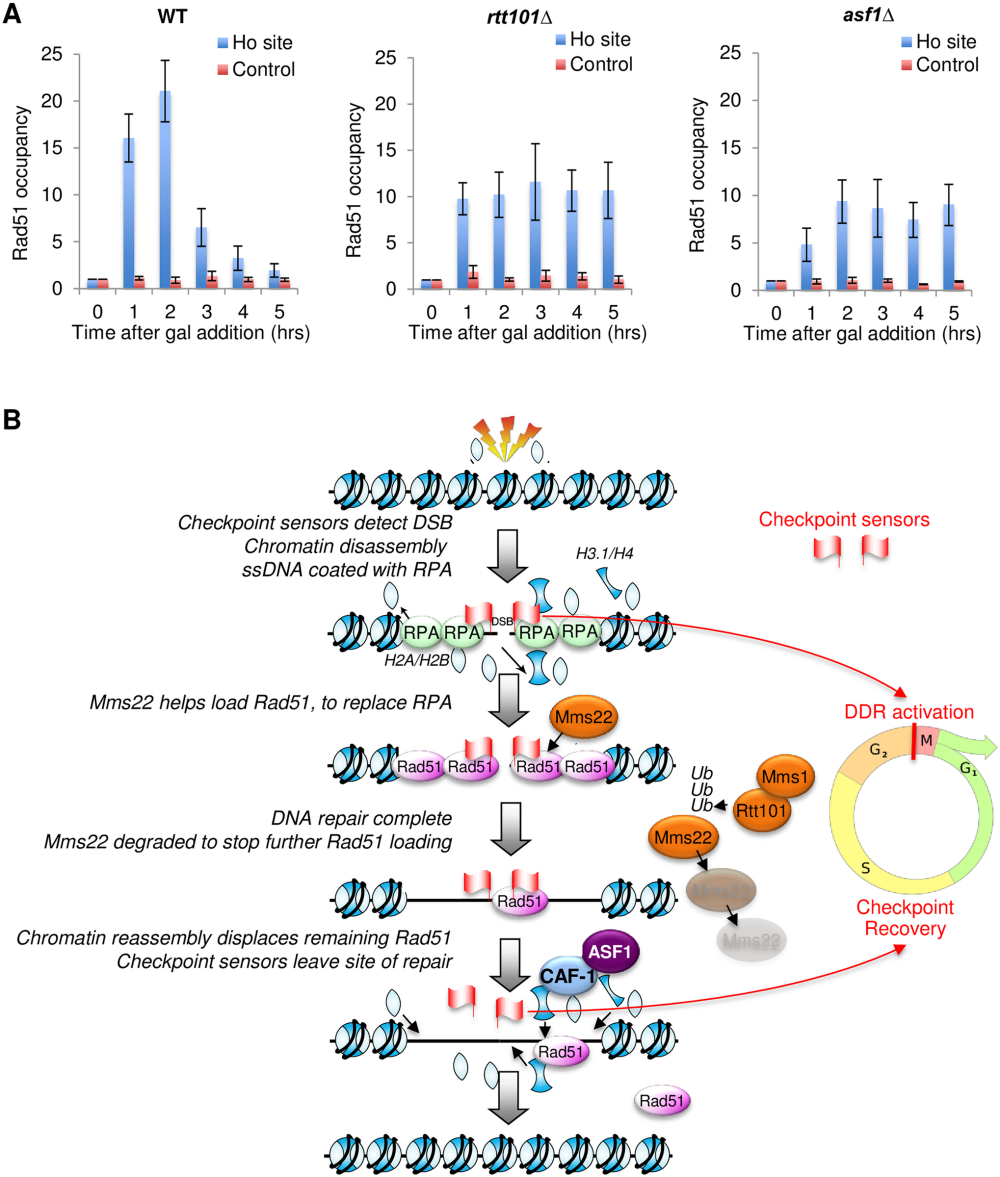
**A**. ChIP analysis of Rad51 levels flanking the DSB site and a TELVIR control region in WT (YMV045), *asf1*Δ (JKT200) and *rtt101*Δ (CCY019) strains. Data are the average and standard deviation of three independent experiments. The experiments in **B**. Schematic summarizing the results of this study, as explained in the text.

The lack of Rad51 binding around a DSB in the *mms22* mutant (Fig 5C) could be for various reasons: 1. failure to load Rad51, 2. failure to remove RPA, or 3. indirect reasons, such as defective expression of Rad51, or channeling the lesion into the break induced replication pathway, which does not require Rad51. To differentiate between these possibilities, we examined Rad51 recruitment to a DSB in the *rtt101* mutant that is unable to degrade all Mms22 after DSB repair. Consistent with a role of Mms22 in loading Rad51 in yeast, we observed persistent recruitment of Rad51 to the vicinity of the HO site in the *rtt101* mutant, even at late time points at which DSB repair was complete (Fig 6A).

Noteworthy, we also observed persistent Rad51 presence at the DSB in the *asf1* mutant (Fig 6A). Given that DSB repair is complete in an *asf1* mutant as detected by southern blotting analysis and semi-quantitative PCR over the break [24] [35] we asked whether Rad51 may remain on the ssDNA overhangs. Repair by SSA involves extensive 5′-to-3′ resection of the broken DNA ends, which leaves 3′-ended ssDNA tails that are coated with Rad51. After homologous regions anneal, these flaps can still persist, until they are cleaved off with Rad1-10 [42]. Therefore we asked whether ssDNA overhangs persist in the *asf1* mutant, and found that cleavage of the ssDNA overhangs was as effective as wild type, albeit delayed by approximately 2 hours. All these data suggest that Asf1-mediated chromatin assembly may also contribute to physical displacement of some of the Rad51 from the site of DSB repair, in order to promote checkpoint recovery (S5 Fig). Taken together, these data indicate that Rad51 is remaining on dsDNA at the sites of repair when Mms22 is not degraded and when chromatin assembly is blocked.

## Discussion

The mechanism whereby chromatin assembly promotes checkpoint recovery was unknown. The findings here suggest that the role of chromatin assembly in checkpoint recovery after DSB repair involves the removal of excess Rad51, and the removal of the checkpoint sensors Ddc1 and Ddc2 from the site of DNA repair. We also provide evidence for a parallel mechanism of Rad51 removal from dsDNA after repair, via the Rtt101^Mms1^-mediated ubiquitylation and degradation of Mms22 to promote checkpoint recovery after DSB repair (Fig 6B).

Chromatin assembly after DSB repair requires the histone chaperone Asf1 [24]. However, Asf1 does not deposit histones onto the DNA itself, rather it hands them to downstream histone chaperones including CAF-1 and Rtt106 during DNA synthesis, and HIRA in the absence of DNA synthesis [43]. We show here that CAF-1 contributes to chromatin assembly after DSB repair (Fig 1C), consistent with the role of CAF-1 in checkpoint recovery [35]. Noteworthy, there is no defect in DSB repair in the absence of either Asf1 or CAF-1 [35], consistent with our earlier studies [24, 44]. This underscores our conclusion that reduced chromatin assembly after DSB repair is causing a checkpoint recovery defect, rather than persistent checkpoint activation due to a DNA repair defect. The identity of the other histone chaperones that receive the histones from Asf1 to deposit them onto the newly-repaired DNA is not clear, but we have ruled out a role for Rtt106 in this function (Fig 1B). The H3/H4 chaperone FACT may be a candidate for the remainder of the chromatin assembly following DSB repair, given its recently demonstrated role in chromatin assembly after DSB replication [45]. Noteworthy, the role of FACT in chromatin assembly during replication was promoted by H3 K56 acetylation [45], reminiscent of our previous demonstration that a mutation that mimics H3 K56 acetylation, H3 K56Q, could bypass the requirement for Asf1 in chromatin assembly after DSB repair [24]. Likewise, acetylation of H3 K56Ac also increases the affinity of histones for CAF-1 [27], consistent with our finding a role for CAF-1 in promoting chromatin assembly following DSB repair. It will be interesting to test whether FACT is indeed involved in chromatin assembly after DSB repair in the future. Unfortunately, testing whether FACT is also required for checkpoint recovery will be difficult, given that FACT is essential and the conditional mutants are unlikely to survive long enough at the non-permissive temperature to measure checkpoint recovery.

The key remaining question is: how does chromatin reassembly turn off the DNA damage checkpoint? In the *asf1* mutants that cannot assemble chromatin after DSB repair, we find evidence for the persistent presence of Ddc1, Ddc2, and Rad51 at the site of repair. This is reminiscent of the checkpoint recovery defect in *srs2* mutants, where failure to remove Rad51 from ssDNA overhangs leads to the persistent presence of Ddc1 and Ddc2 and hence a defect in checkpoint recovery [22]. However, in the *asf1* mutants, the persistence of Rad51 is not due to it binding to ssDNA overhangs, because ssDNA overhangs are effectively removed in the *asf1* mutant. This suggests that Rad51 is bound to the dsDNA over the repair site in the absence of chromatin assembly after DSB repair. Removal of the checkpoint sensors Ddc1 and Ddc2 from the site of DNA repair after repair is complete is critical for checkpoint recovery, but how this occurs is unknown. Our studies suggest that chromatin assembly after DSB repair functions to displace the checkpoint sensors from the DNA. An alternative explanation for the persistent activation of the DNA damage checkpoint after DNA repair in the *asf1* mutant is that it could be a consequence of the elevated basal level of checkpoint activation in the absence of Asf1. However, this is unlikely to explain the persistent presence of Rad51 at repaired sites in an *asf1* mutant.

Excess Rad51 bound to dsDNA inhibits cell growth [46], presumably via persistent DNA damage checkpoint activation in the absence of DNA damage. Indeed, cells place much effort into removing excess Rad51 from dsDNA, having evolved multiple specialized proteins to do this function. The Rad54 and Rdh54 translocases remove Rad51 from dsDNA *in vitro* [47, 48] and in mitotic cells [46]. There is previous evidence for failure to remove Rad51 from dsDNA causing a defect in checkpoint recovery. Functionally, Rdh54 is required for recovery from cell cycle arrest caused by an induced DSB during mitosis, where the arrest is genetically dependent on *RAD51* [49]. These data all point to the persistent presence of Rad51 on dsDNA being at least partially responsible for the persistent checkpoint activity after DNA repair in mutants unable to assemble chromatin.

It will be interesting to determine whether chromatin assembly is involved in the physical displacement of Rad51 from the site of DSB repair, perhaps working in concert with the translocases Rad54 and Rdh54 to remove Rad51. The persistent presence of Rad51 on the DNA in the absence of Asf1 suggests that the Rad54 and Rdh54 translocases are not sufficient to remove all Rad51 from dsDNA *in vivo*. Indeed, earlier evidence has shown that Rad51 and Rad54 have evolved to function within chromatin [50]. We wondered whether deletion of *RAD51* would fix the checkpoint recovery defect in *asf1* mutants, given that deletion of *RAD51* suppresses the DNA damage sensitivity of the *srs2* mutant, where Srs2 functions to remove Rad51 [18]. However, we found that deletion of *RAD51* is not sufficient to rescue the sensitivity of the *asf1* mutant to the HO lesion in the SSA system (data not shown). Furthermore, overexpression of Rad54 or Rdh54 from a high copy plasmid was also not sufficient to suppress the DNA damage sensitivity of the *asf1* mutant (data not shown). These results suggest that chromatin assembly mediated removal of Rad51 from dsDNA is not the only function of Asf1 during checkpoint recovery. This is not surprising given the requirement for Asf1 in H3 K56 acetylation [51], chromatin assembly [25], efficient PCNA ubiquitylation [52] and the maintenance of genome stability [53]. Furthermore, upon deletion of *RAD51*, the HO lesion generated is repaired by break-induced replication (BIR) [54, 55], which is a *RAD51*-independent repair mechanism that requires all of the replication factors except those specific for pre-replication complex assembly [56]. The fact that deletion of *RAD51* does not suppress the DNA damage sensitivity of the *asf1* mutant to the HO lesion suggests that Asf1 likely also contributes to events during BIR.

We have uncovered a novel role for Rtt101^Mms1^ in checkpoint recovery after repair of a single DSB lesion. Specifically, Rtt101^Mms1^ is required for checkpoint recovery, but not for chromatin assembly, nor for DSB repair. This is in contrast to the situation during replication, where Rtt101^Mms1^ promotes chromatin assembly by ubiquitylating the newly-synthesized histone H3 to promote it being transferred from Asf1 to downstream histone chaperones [57]. Deletion of *RTT101* or *MMS1* leads to a defect in chromatin assembly after DNA replication [57], while chromatin assembly after DSB repair is intact upon deletion of *RTT101* and *MMS1* (Fig 3E). Therefore, the role of Rtt101^Mms1^ in survival after DSB repair appears more likely to be related to regulating the levels of the substrate adaptor protein Mms22, than promoting chromatin assembly.

The Haber group recently reported that Asf1 and Rtt101 are required for checkpoint recovery, but in their hands they only had this role when they induced two DSBs [58], not one DSB as is the case in our studies [24] Fig 3. This may be related to the fact that the Haber lab only observed defective checkpoint recovery in response to a single DSB when they remove both Asf1 and CAF-1 at the same time, but not with the individual mutations [35] as is the case in our hands [24]. This functional redundancy between Asf1 and CAF-1 [35] was surprising given that Asf1 and CAF-1 function in a linear pathway during chromatin assembly [43]. For unclear reasons, it would appear that their strains or experimental procedures lead to less stringent requirements for checkpoint recovery than in our experiments.

The idea that Asf1 mediates checkpoint recovery in a histone chaperone-independent manner has been proposed recently. This was based on a histone mutation that weakens the Asf1-histone interaction being able to partially suppress the checkpoint recovery defect of *rtt109* and *rtt101* mutants [58]. This work suggested that histone-free Asf1 binding to Rad53 may promote Rad53 dephosphorylation, through unclear mechanisms. Although the effect on chromatin assembly after DSB repair was not tested, histone mutations that weaken the histone-Asf1 interaction are likely to actually promote chromatin assembly *per se* not block it, given that histone modifications that weaken the Asf1-histone interaction such as Rtt101^Mms1^-mediated H3 Ub and Rtt109-mediated K56 acetylation, promote the transfer of histones from Asf1 to the downstream histone chaperone CAF-1 to enhance chromatin assembly [27, 57]. This ability of the histone mutant that weakens Asf1-histone interaction to partially suppress the checkpoint recovery defect in the *rtt101* mutant is also perplexing [58] given that the *rtt101* mutant is fully able to assemble chromatin after DSB repair (Fig 3E). Furthermore, a histone-chaperone independent model of Asf1 in checkpoint recovery [58] would not explain how the cell overcomes the persistent presence of the checkpoint sensors Ddc1 and Ddc2 at the site of repair, which presumably persistently signals for Rad53 phosphorylation, in the *asf1* mutants (Fig 2). A more thorough test of a potential histone chaperone-independent role of Asf1 in checkpoint recovery would be to use the Asf1 V94R mutant that is totally unable to bind to histones.

We have found that the yeast substrate-specific adaptor Mms22 plays a role in DSB repair *per se*, facilitating the loading of Rad51 onto DNA. This repair defect was seen at the level of greatly reduced DNA religation (Fig 5B) and failure to recruit significant amounts of Rad51 to the DSB (Fig 5B). This lead to damage sensitivity to the induced HO break that is repaired by SSA (Fig 3A and 3B), cell cycle delay and persistent Rad53 phosphorylation (Fig 5B) in the absence of Mms22. A previous genetic analysis measuring HO endonuclease-stimulated unequal sister chromatid exchange found no significant role for either Mms22 or Mms1 in this process [59]. Similarly, analysis of repair of an HO lesion using a homologous region on another chromosome found a significant, but not striking, defect in the absence of Mms22 [59]. The reason why we see a greater requirement for Mms22 in DSB repair than this previous study may be related to the requirement for 5kb of DNA resection for the repair assay that we are using in our study. In agreement with this idea, our data indicate a role for Mms22 in loading the single-strand binding protein Rad51 onto the resected DNA. Consistent with our findings in yeast, human Mms22 plays a role in loading Rad51 onto ssDNA, mediated by a direct interaction between Mms22 and Rad51 [31, 60].

Our data suggest that the Rtt101^Mms1^ mediated-ubiquitylation and degradation of Mms22 appears to be important for ending the persistent loading of Rad51 onto dsDNA after DSB repair. There must be a mechanism for activation of Rtt101^Mms1^ to ubiquitylate Mms22, but only once DSB repair is complete. It will be interesting to determine how activation of Rtt101^Mms1^ is triggered after DSB repair, presumably requiring either Rtt101 neddylation and / or ubiquitylation after DSB repair [61]. Our studies may also provide some molecular insight into the role of Rtt101^Mms1^ and Mms22 during DNA replication fork restart. For example, the role of Mms22 during DNA replication fork restart may also turn out to be in promoting Rad51 loading, because recently Mms22 has been proposed to direct stalled replication forks to be resolved via an HR mechanism [62]. Future analyses will examine whether this is indeed the case. Also, the mechanism whereby retention of Rad51 leads to persistent presence of the checkpoint sensors in the absence of DNA damage is unclear. Studies in this area will help elucidate the elusive question of how checkpoint sensor proteins are removed from the site of DNA damage, in order to allow the cell to resume the cell cycle after DNA repair.

## Supporting information

S1 Fig Analysis of input to H3 histone ChIPs shows that repair is efficient in strains lacking CAC2 and ASF1. Shown are input DNA adjacent to the HO lesion at MAT, normalized to a control region where no cutting occurs in strain WT (YMV045), *asf1*Δ (JKT200) and *cac2*Δ (JLY078) strains. The HO lesion was induced by adding galactose at time 0.
(TIFF)

S2 Fig The levels of the Rad53 phosphatase Ptc2 that is required for checkpoint recovery is normal in asf1 and rtt101 mutants. WT (YMV045), *asf1*Δ (JKT200), *rtt101*Δ (CCY019) and *ptc2*Δ (63) strains were used.
(TIFF)

S3 Fig A. Chromatin fractionation assay of RTT101-MYC in wild-type strain (LWY012) and *asf1*Δ (LWY003). Immunoblotting of Histone 3 and RTT101-MYC in various fractions: T, total cell extracts; P, pellet; and S, supernatant after with or without Zeocin treatment for 2 hours.
(TIFF)

S4 Fig Calculation of colony formation from single unbudded cells following the indicated length of times of growth on galactose-containing plates in *mms22*Δ (CCY024). Error bars represent standard deviation calculated from three independent experiments. The experiment shown was done in parallel with those in Fig 3D.
(TIFF)

S5 Fig Analysis of single-stranded DNA overhangs, using strains WT (YMV045), *asf1*Δ (JKT200) and *rad1*Δ (LWY061). (TIFF)
