# Supplementary figures and images for: Delineation of the role of chromatin assembly and the Rtt101Mms1 E3 ubiquitin ligase in DNA damage checkpoint recovery in budding yeast
Source: PLoS One. 2017 Jul 27;12(7):e0180556. doi: 10.1371/journal.pone.0180556 (PMC5531559; doi:10.1371/journal.pone.0180556)

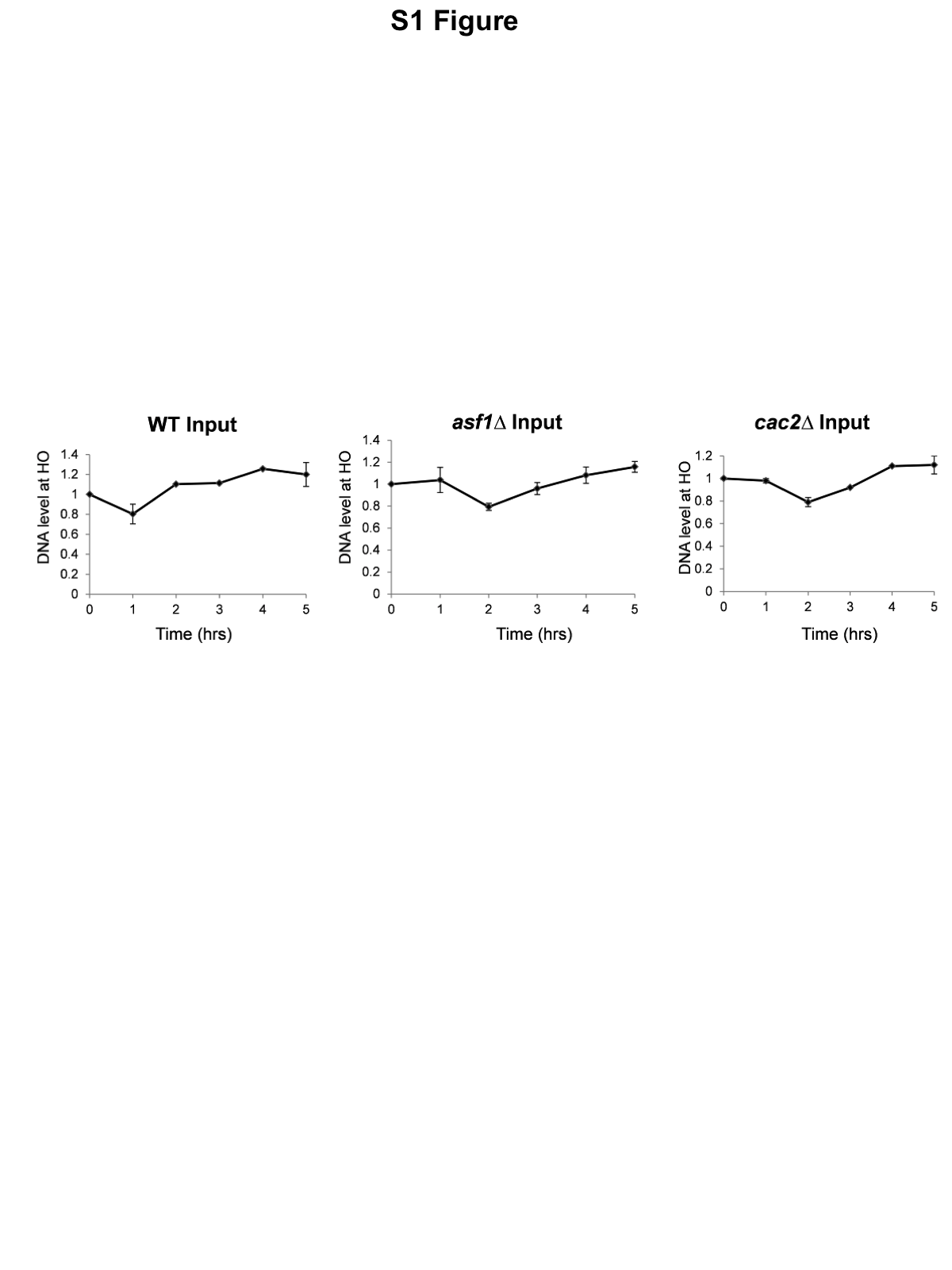

Supplement: S1 Fig — Shown are input DNA adjacent to the HO lesion at MAT, normalized to a control region where no cutting occurs in strain WT (YMV045), asf1Δ (JKT200) and cac2Δ (JLY078) strains. The HO lesion was induced by adding galactose at time 0. (TIFF) [file pone.0180556.s001.tiff]

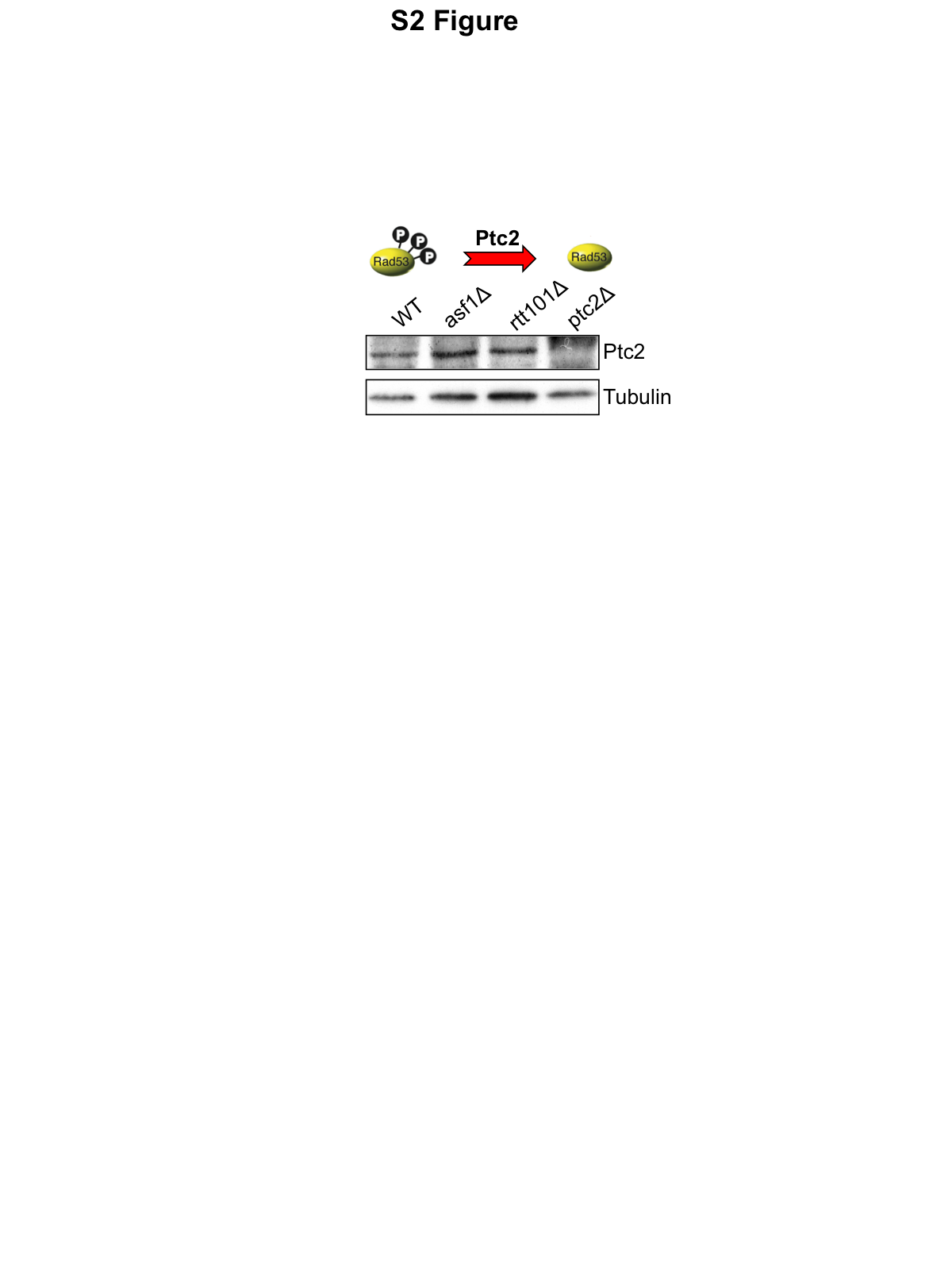

Supplement: S2 Fig — WT (YMV045), asf1Δ (JKT200), rtt101Δ (CCY019) and ptc2Δ (63) strains were used. (TIFF) [file pone.0180556.s002.tiff]

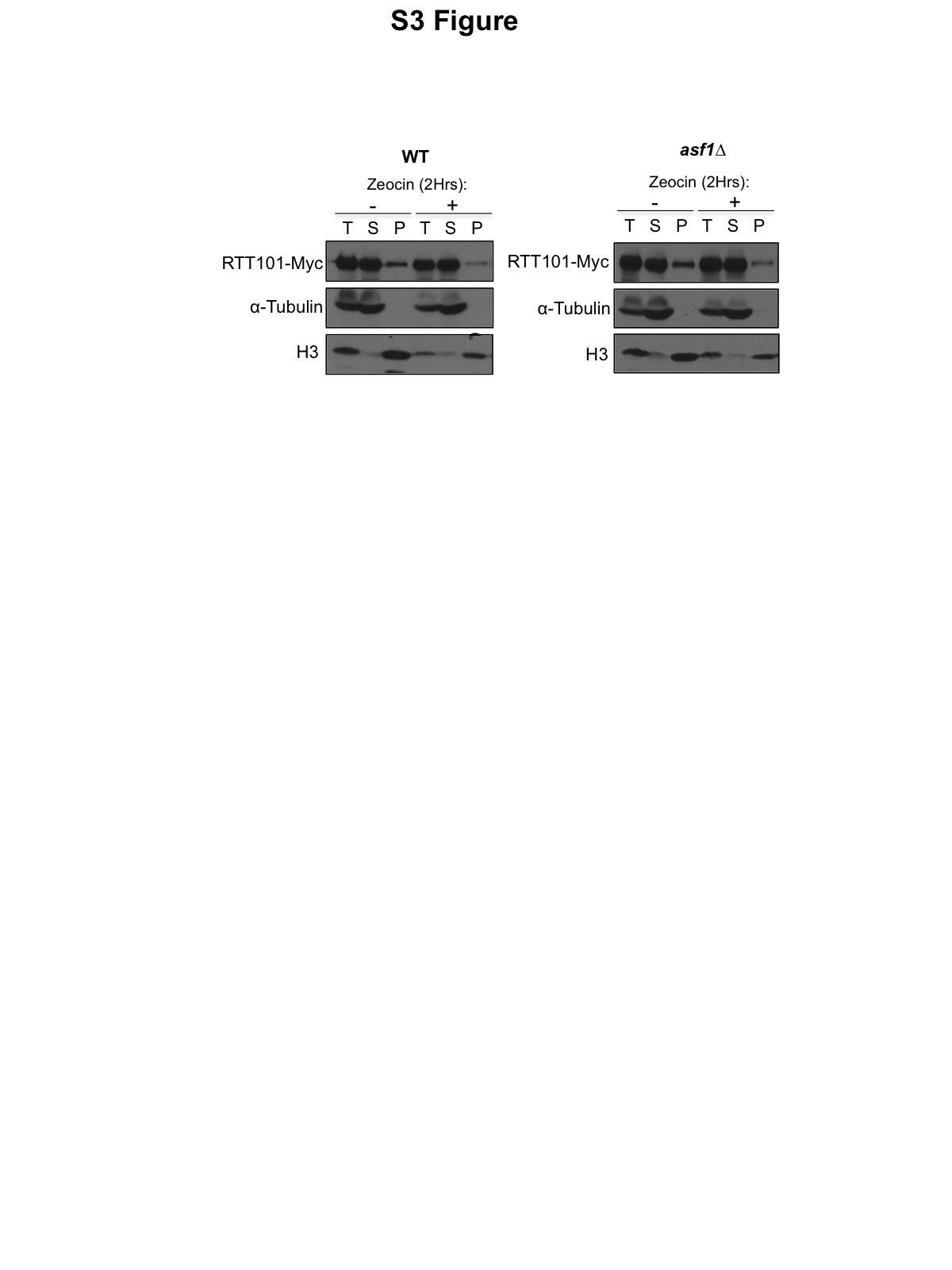

Supplement: S3 Fig — Immunoblotting of Histone 3 and RTT101-MYC in various fractions: T, total cell extracts; P, pellet; and S, supernatant after with or without Zeocin treatment for 2 hours. (TIFF) [file pone.0180556.s003.tiff]

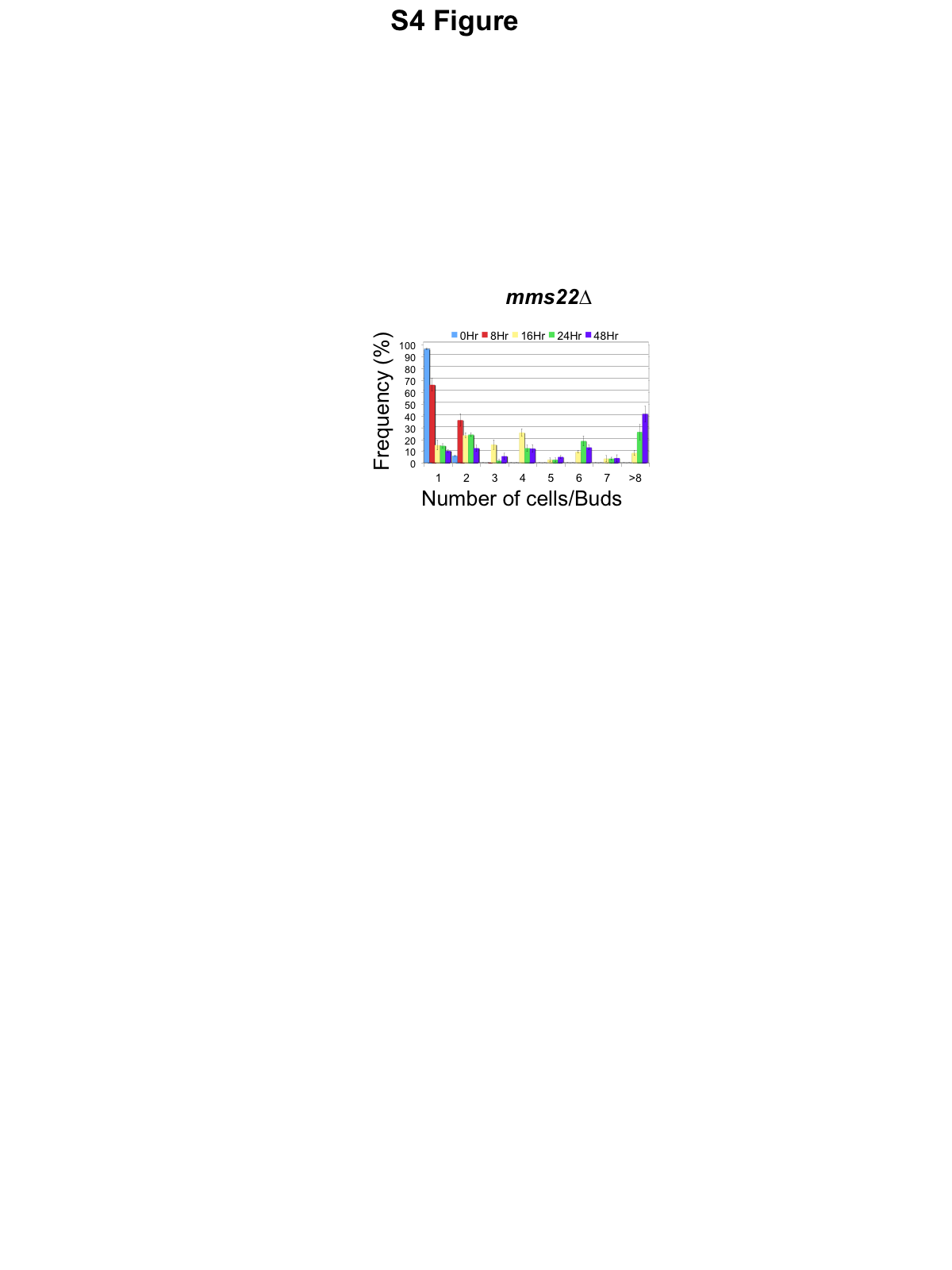

Supplement: S4 Fig — Error bars represent standard deviation calculated from three independent experiments. The experiment shown was done in parallel with those in Fig 3D. (TIFF) [file pone.0180556.s004.tiff]

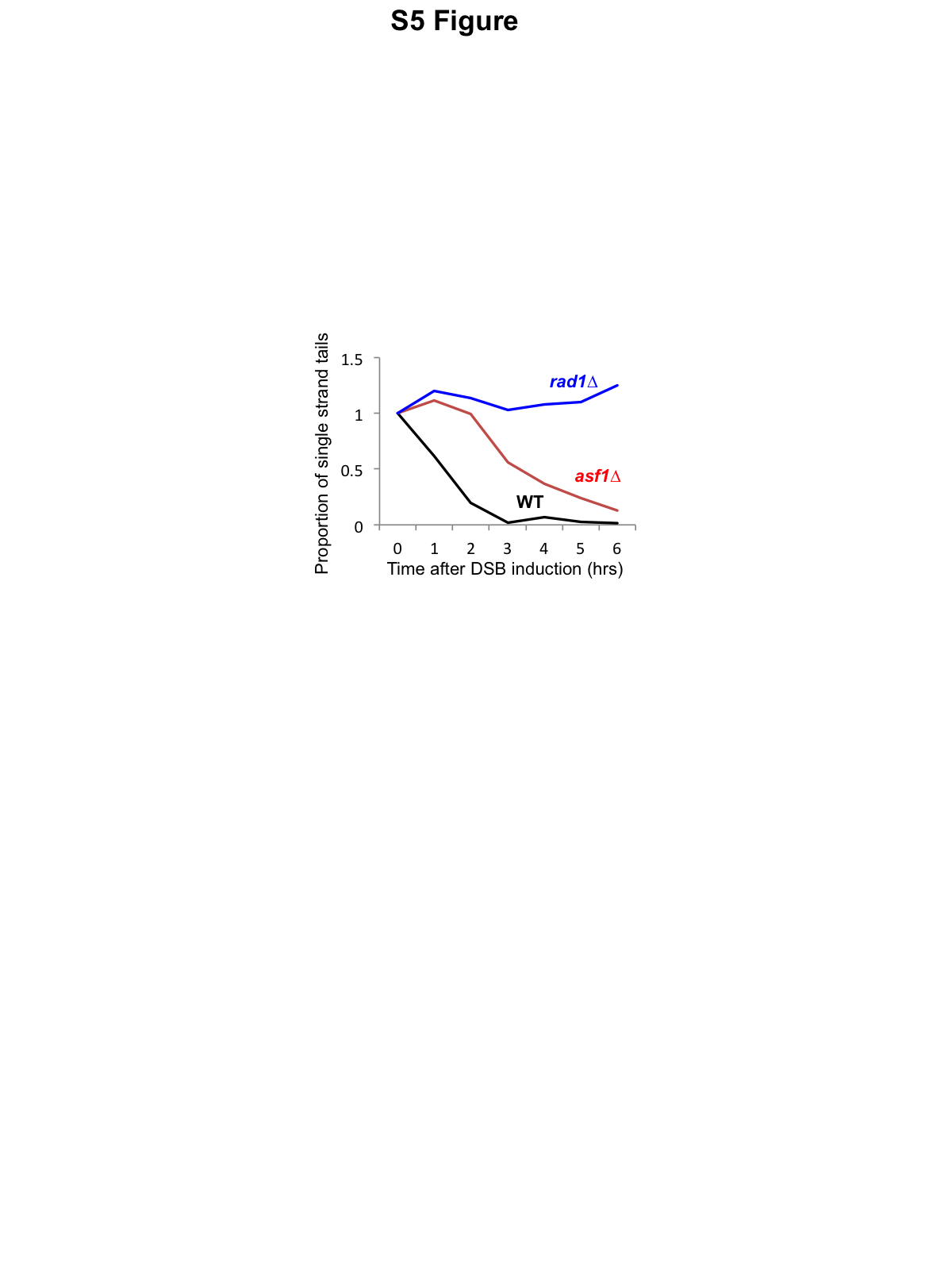

Supplement: S5 Fig — (TIFF) [file pone.0180556.s005.tiff]
